# Author Correction: Structure of the endosomal CORVET tethering complex

**DOI:** 10.1038/s41467-024-53199-0

**Published:** 2024-10-10

**Authors:** Dmitry Shvarev, Caroline König, Nicole Susan, Lars Langemeyer, Stefan Walter, Angela Perz, Florian Fröhlich, Christian Ungermann, Arne Moeller

**Affiliations:** 1 Department of Biology/Chemistry, Structural Biology Section, Osnabrück University, 49076 Osnabrück, Germany; 2 Department of Biology/Chemistry, Biochemistry Section, Osnabrück University, 49076 Osnabrück, Germany; 3 Center of Cellular Nanoanalytics Osnabrück (CellNanOs), Osnabrück University, 49076 Osnabrück, Germany; 4 Department of Biology/Chemistry, Bioanalytical Chemistry Section, Osnabrück University, 49076 Osnabrück, Germany

**Keywords:** Cryoelectron microscopy, Cryoelectron microscopy, Membrane fusion, Biophysics

Correction to: *Nature Communications* 10.1038/s41467-024-49137-9, published online 19 June 2024

The original version of this Article contained an error in Fig. 6, in which “Rab7/Ypt7” in the inset illustrating the CORVET tethering complex was mislabelled and should read “Rab5/Vps21”. The correct version of Fig. 6 is:
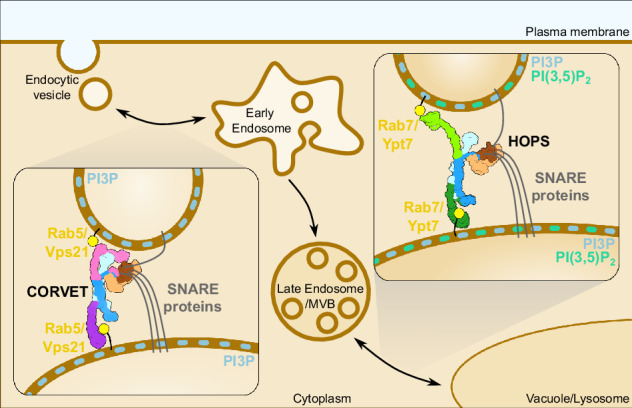


which replaces the previous incorrect version:
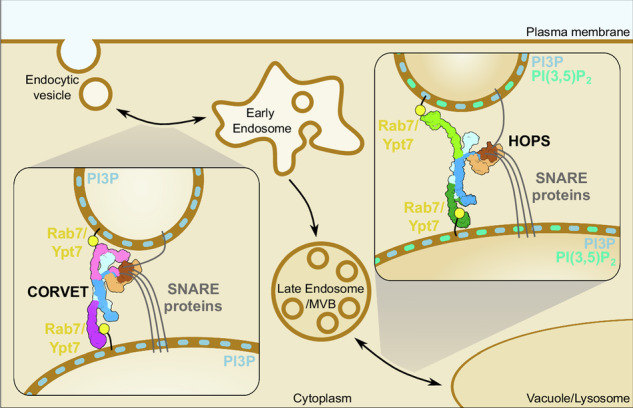


This has been corrected in both the PDF and HTML versions of the Article.

