# Supplementary material for: Structure of the endosomal CORVET tethering complex
Source: Nat Commun. 2024 Jun 19;15:5227. doi: 10.1038/s41467-024-49137-9 (PMC11187117; doi:10.1038/s41467-024-49137-9)
Supplement: Supplementary file 1 — Supplementary Information [file 41467_2024_49137_MOESM1_ESM.pdf]

Supplementary Data for:

Structure of the endosomal CORVET tethering complex

Dmitry Shvarev[1]†, Caroline König[2]†, Nicole Susan[2]†, Lars Langemeyer[2,3], Stefan Walter[3], Angela Perz[2], Florian Fröhlich[3,4], Christian Ungermann[2,3]*, Arne Moeller[1,3]*

[1]Osnabrück University, Department of Biology/Chemistry, Structural Biology section; 49076 Osnabrück, Germany.

[2]Osnabrück University, Department of Biology/Chemistry, Biochemistry section; 49076 Osnabrück, Germany.

[3]Osnabrück University, Center of Cellular Nanoanalytics Osnabrück (CellNanOs); 49076 Osnabrück, Germany.

[4]Osnabrück University, Department of Biology/Chemistry, Bioanalytical Chemistry section; 49076 Osnabrück, Germany.

*Corresponding authors. Email: cu@uos.de (C.U.), arne.moeller@uos.de (A.M.)

† These authors contributed equally to this work.

Supplementary Figures

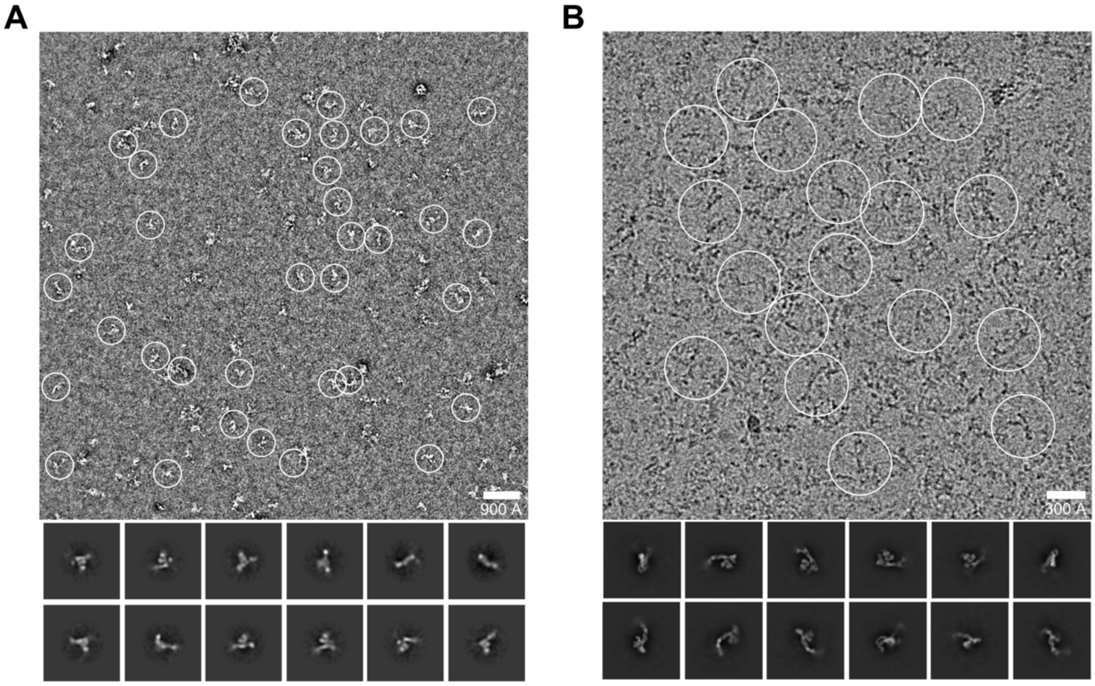

Supplementary Figure 1. **Single particle analysis of wild-type CORVET.** Representative negative stain **(A)** and cryo-EM **(B)** micrographs and 2D class averages (bottom). Box size used for particle extraction: 799 Å (negative stain), 815 Å (cryo-EM).

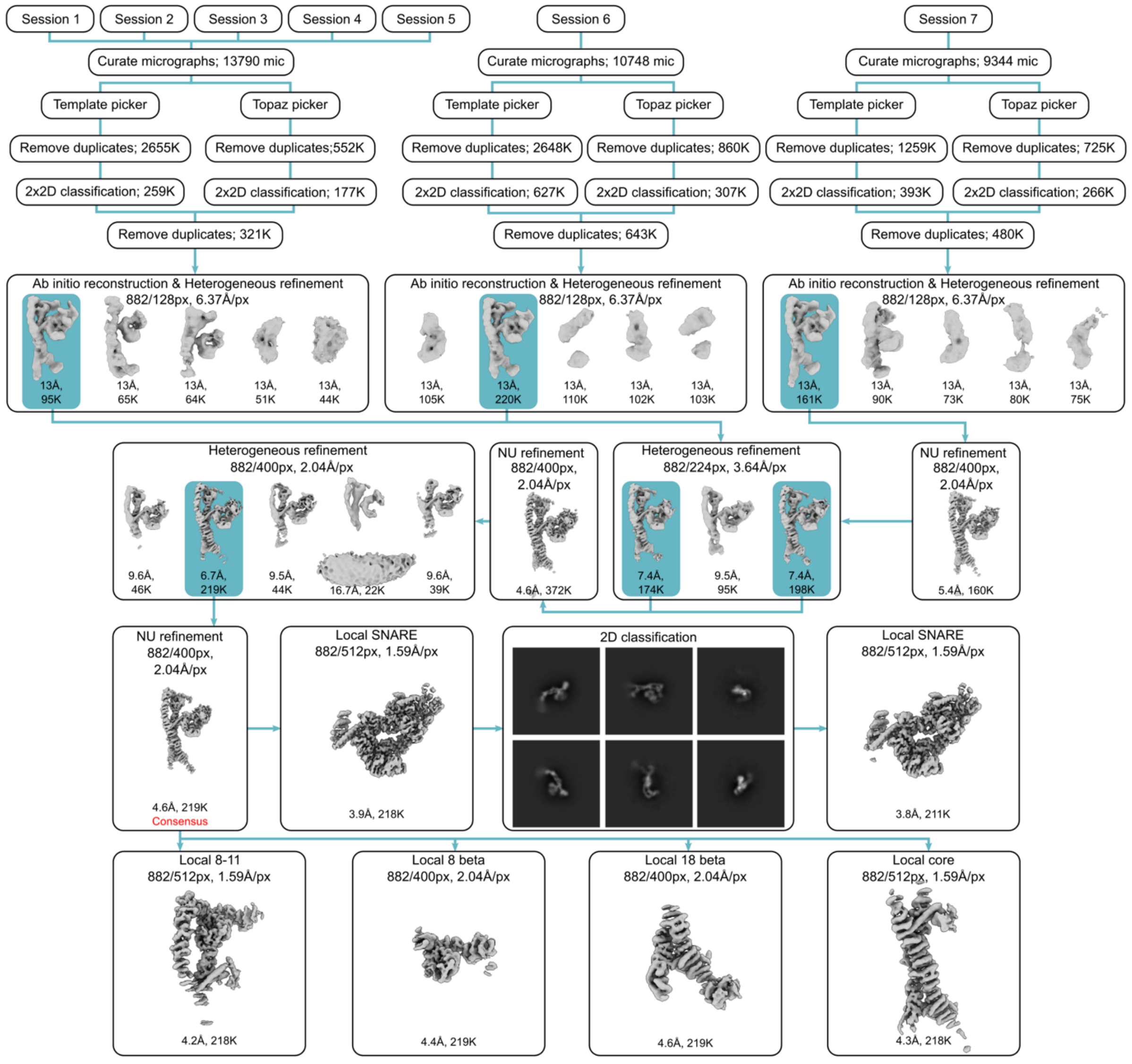

Supplementary Figure 2. Cryo-EM data processing workflow of wild-type CORVET. The main steps of the data processing pipeline performed in cryoSPARC are shown. Box sizes (full/cropped) in pixels, resulting pixel size, number of particles, and resolution achieved are shown for each map shown.

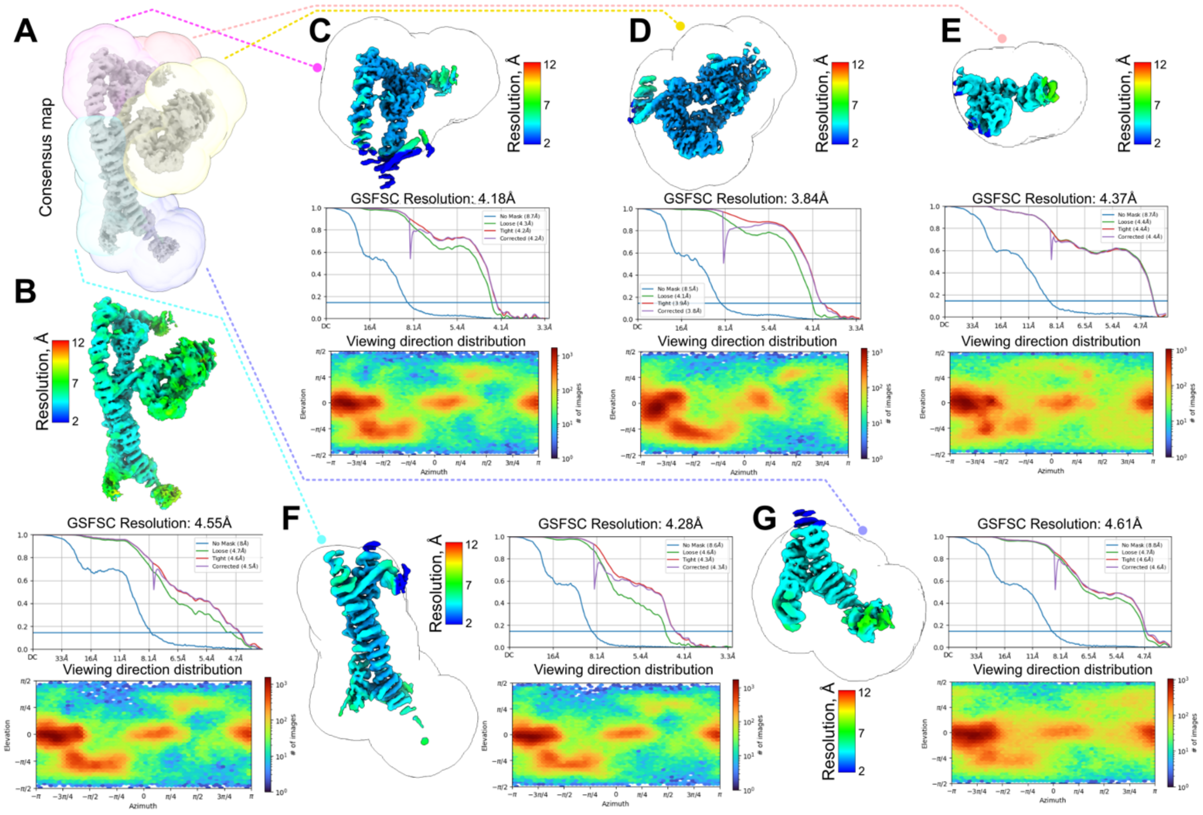

Supplementary Figure 3. **Local refinement approach and cryo-EM data validation. A,** Masks (semi-transparent) used for local refinement of the consensus map fragments. **B-G,** Local resolution estimation, Gold Standard Fourier shell correlation (GSFSC) curve, and angular distribution heatmap plot generated in cryoSPARC for the consensus map **(B)** and final local refinement maps **(C-G)**.

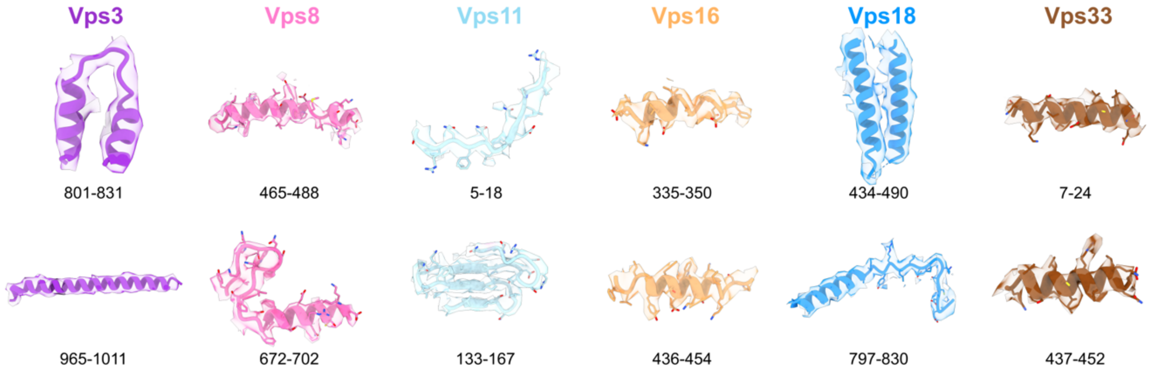

Supplementary Figure 4. **Cryo-EM quality.** Model/map fit of selected areas within each subunit of CORVET. Coloring is as in Figure 1.

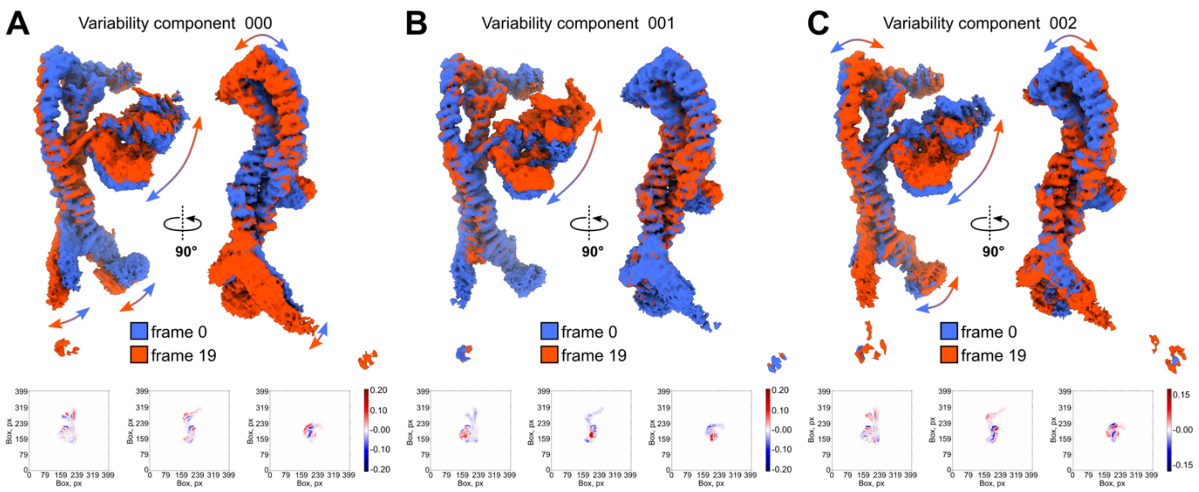

Supplementary Figure 5. **3D variability analysis of CORVET. A-C,** 3D density maps (top) and map 2D projections (bottom) at negative (blue) and positive (red) positions along each variability component are shown.

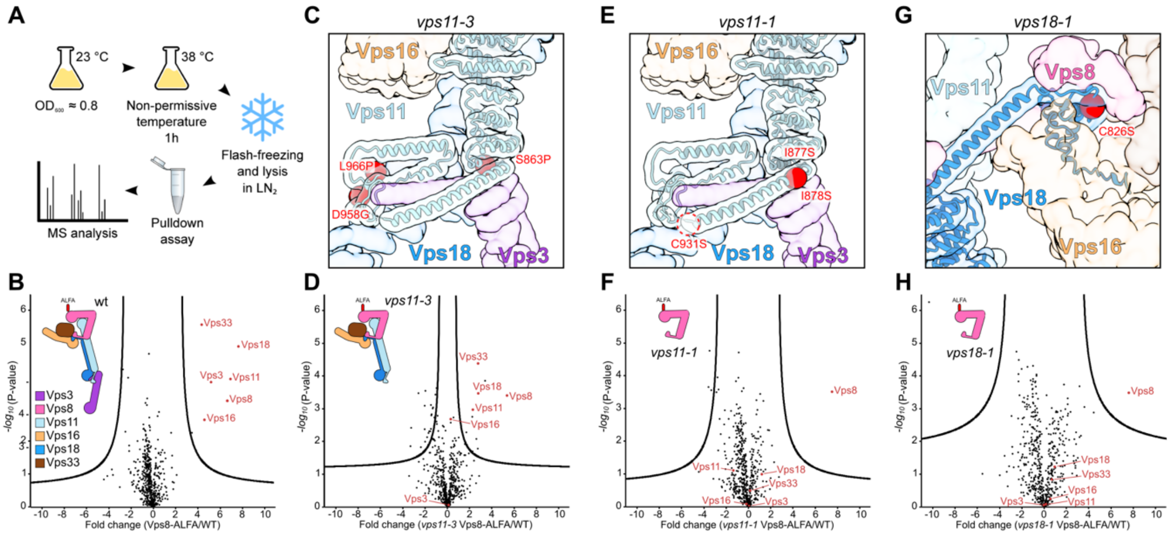

Supplementary Figure 6. **Analysis of RING finger domains role in the structural integrity of CORVET. A,** Scheme of the experiment setup. **B,D,F,H**, Mass spectrometry analysis of Vps8 purified via the ALFA tag from the indicated strains and enriched proteins (red dots). Results of purification from wt (**B**), *vps11-3* (**D**), *vps11-1* (**F**), and *vps18-1* (**H**) cells. **C,E,G**, Positions of the mutated residues (red spheres) in the respective mutants of the core subunits Vps11 or Vps18 (shown in ribbon representation) in the context of the CORVET structure (semi-transparent envelope). CORVET subunits are colored as in as in Figure 1.

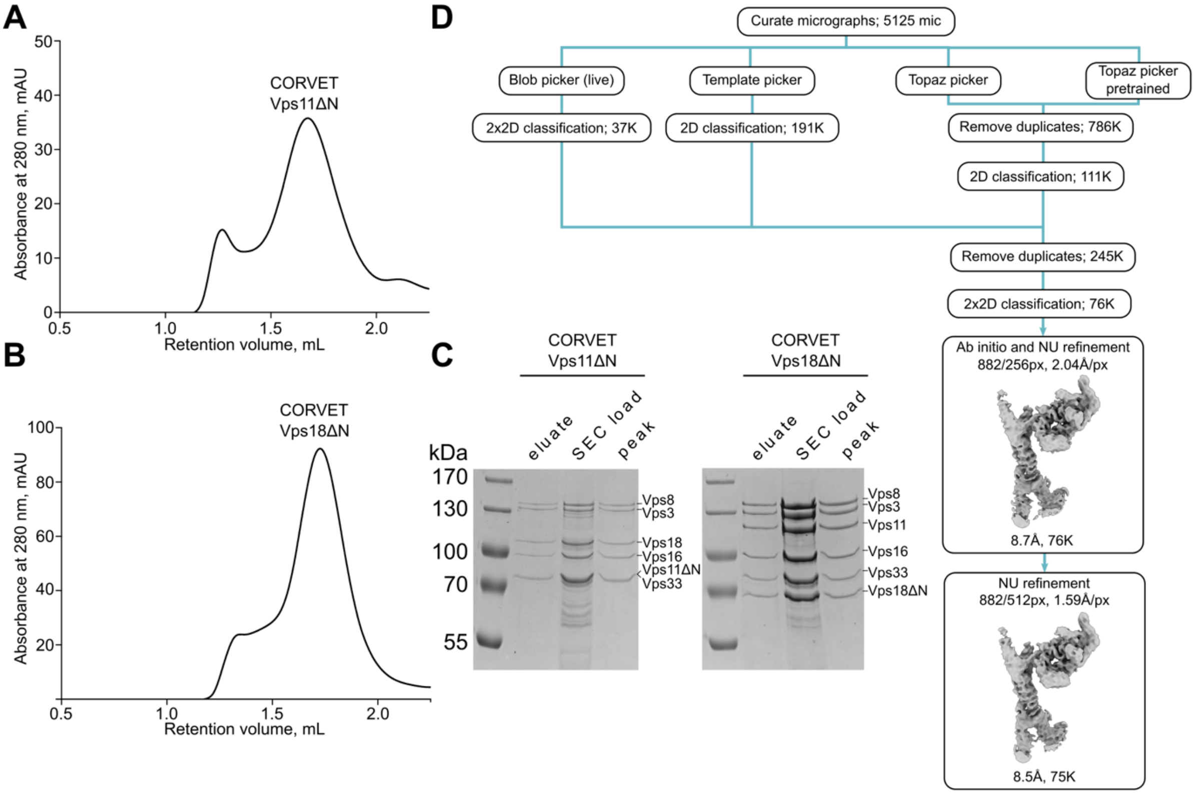

Supplementary Figure 7. **Purification, biochemical analysis and cryo-EM workflow of the CORVET Vps11ΔN mutant complex. A,** Size exclusion chromatography (SEC) of the affinity-purified CORVET Vps11ΔN. **B**, SEC of the affinity-purified CORVET Vps18ΔN. **C**, SDS-PAGE analysis of purified CORVET Vps11ΔN and Vps18ΔN mutant complexes. Protein samples from affinity purification (eluate) and before and after SEC are shown. n=3 independent experiments were performed. **D**, The main steps of the data processing pipeline of the cryo-EM CORVET Vps11ΔN sample performed in cryoSPARC are shown. Box sizes (full/cropped) in pixels, resulting pixel size, number of particles, and resolution achieved are shown for each map shown.

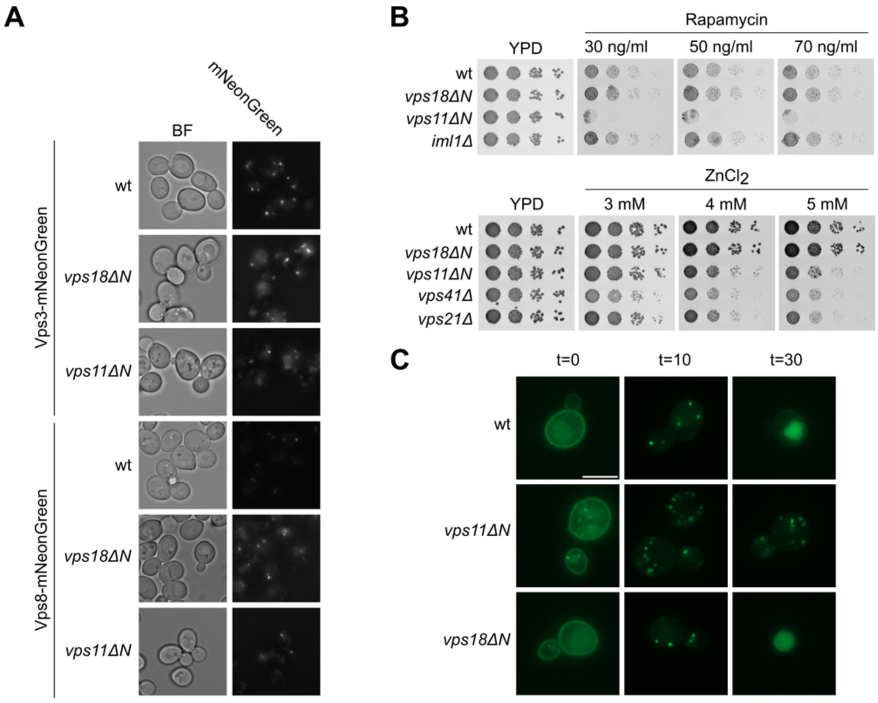

Supplementary Figure 8. **Functional analysis of CORVET mutant strains. A**, Localization of Vps3 and Vps8 in Vps11 and *vps18ΔN* mutants. Vps3 or Vps8 were genomically tagged with mNeonGreen. Cells were grown to log phase and visualized by fluorescence microscopy. **B**, Growth test assays. Cells were grown to the same OD_{600} and spotted in serial dilutions on yeast extract peptone dextrose medium (YPD) without or with Rapamycin or $ZnCl_2$. Plates were grown at 30°C for 1-2 days before imaging. **C,** Mup1 uptake assay. Mup1 was C-terminally tagged with msGFP2 in wild-type, *vps11ΔN* and *vps18ΔN* cells. Cells were grown in synthetic medium lacking methionine and analyzed by fluorescence microscopy (t=0), before shifting to methionine-containing medium for 10 min (t=10) and 30 min (t=30). Scale bar: 5 µm. Quantification is shown in Figure 5. For microscopy and growth assays, n=3 independent experiments were performed.

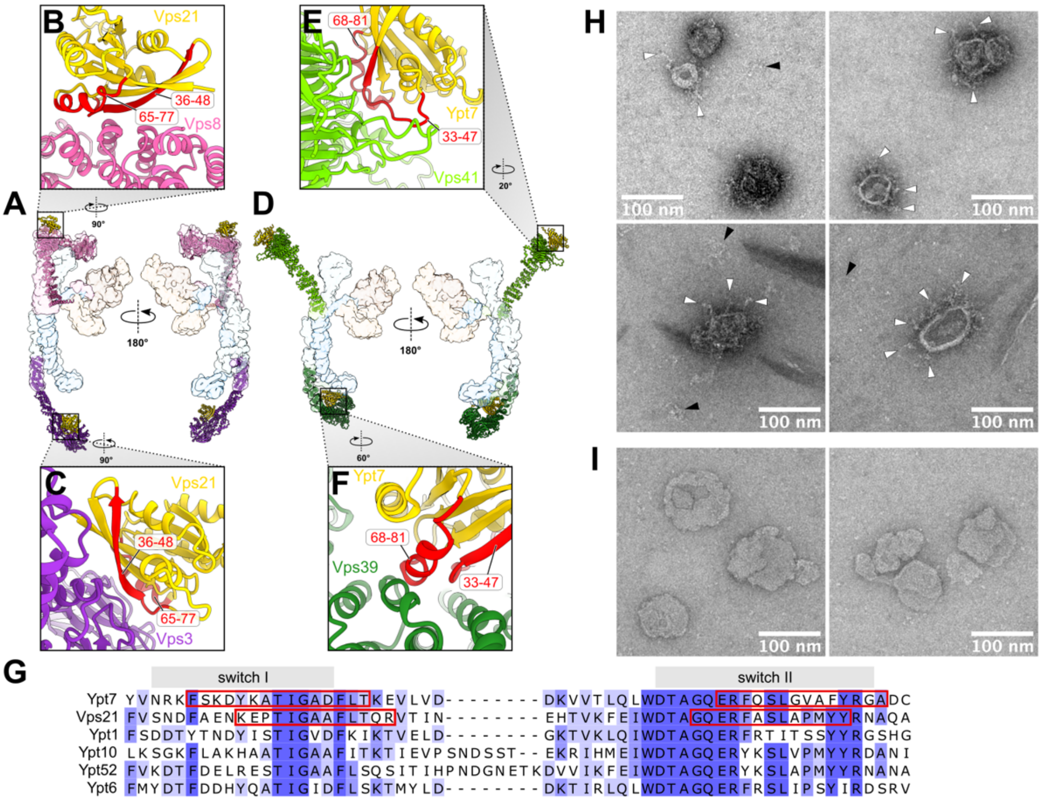

Supplementary Figure 9. Analysis of CORVET interactions with Rab GTPases and membranes. A, Alphafold models Vps8 (pink) and Vps3 (violet) complexed with the Rab GTPase Vps21 (yellow) fitted into the structure of CORVET (this study, semi-transparent envelope) viewed from two sides. **B, C**, Close-up views at the predicted Vps8-Vps21 (**B**) and Vps3-Vps21 (**C**) interfaces. Secondary structural elements of Vps21 involved in the interface are indicated. **D**, Alphafold models Vps41 (light green) and Vps39 (dark green) complexed with the Rab GTPase Ypt7 (yellow) fitted into the structure of HOPS (PDB:7ZU0, semi-transparent envelope) viewed from two sides. **E, F**, Close-up views at the predicted Vps41-Ypt7 (**E**) and Vps39-Ypt7 (**F**) interfaces. Residues of Ypt7 and Vps21 involved in the predicted interface are colored red. CORVET subunits are colored as in as in Figure 1. **G**, Sequence alignment of representative Rab proteins from yeast including Vps21 and Ypt7. The conserved switch regions of Rab proteins are labelled. Red frames highlight residues mapped onto the predicted interaction sites in the structural models (panels **B**, **C**, **E**, **F**). **H,I,** Negative stain EM analysis of Rab5(Vps21)-loaded liposomes containing 10 mol % phosphatidylinositol-3-phosphate (PI3P) incubated with CORVET (**H**) or without (**I**).

Supplementary Tables

Supplementary Table 1. Yeast strains used in the study.

**Strain**	**Genotype**	**Source**
SEY6210	MAT alpha *leu2-3,112 ura3-52 his3-Δ200 trp-Δ901 lys2-801 suc2-Δ9 GAL*	Reggiori Lab
CUY2489	MAT alpha *his3-Δ200 leu2-Δ0 lys2-Δ0 met15-Δ0 trp1-Δ63 ura3-Δ0 VPS11::HIS3-GAL1pr VPS16::NatNT2-GAL1pr VPS18::KanMX-GAL1pr*	1
CUY13053	MATa *his3-Δ200 met15-Δ0 trp1-Δ63 ura3-Δ0 VPS8::TRP1-GAL1pr VPS3::HIS3-GAL1pr VPS33::KanMX-GAL1pr VPS8::HphNT1-FLAG*	This study
CUY13050	MATa *his3-Δ200 met15-Δ0 trp1-Δ63 ura3-Δ0 VPS41::TRP1-GAL1pr VPS39::KanMX-GAL1pr VPS33::HIS3-GAL1pr VPS41::HphNT1-FLAG*	3
CUY13080	CUY2489xCUY13050	3
CUY13081	CUY2489xCUY13053	This study
CUY14313	MAT alpha *his3-Δ200 leu2-Δ0 lys2-Δ0 met15-Δ0 trp1-Δ63 ura3-Δ0 VPS11Δaa1-349::URA3-GAL1Pr VPS16::NatNT2-GAL1pr VPS18::KanMX-GAL1pr x*<br>MATa *his3-Δ200 leu2-Δ0 met15-Δ0 trp1-Δ63 ura3-Δ0 VPS8::TRP1-GAL1pr VPS3::HIS3-GAL1pr VPS8::HphNT1-FLAG VPS33::KanMX-GAL1pr*	This study
CUY14318	MATa *his3-Δ200 met15-Δ0 trp1-Δ63 ura3-Δ0 VPS8::TRP1-GAL1pr VPS33::URA3-GAL1pr Vps3::HIS3-GAL1pr*	This study
CUY13750	MAT alpha *his3-Δ200 leu2-Δ0 lys2-Δ0 met15-Δ0 trp1-Δ63 ura3-Δ0 VPS11::HIS3-GAL1pr VPS16::kanMX-GALpr1 VPS18Δaa1-335::TRP1-GAL1pr VPS18::FLAG-HphNT1*	3
CUY14325	CUY14318xCUY13750	This study
CUY2742	Mat alpha *vps11-1 leu2-3,112 ura3-52 his3-Δ200 trp1-Δ901 lys2-801 suc2-Δ9 VPS11-1::HIS3-HA*	2
CUY2743	Mat alpha *vps11-3 leu2-3,112 ura3-52 his3-Δ200 trp1-Δ901 lys2-801 suc2-Δ9*	2
CUY2744	Mat alpha *vps18-1 leu2-3,112 his3-Δ200 trp1-Δ901 lys2-801 suc2-Δ9*	2
CUY13541	MAT alpha *leu2-3,112 ura3-52 his3-Δ200 trp-Δ901 lys2-801 suc2-Δ9 VPS8::ALFA-HphNT1*	This study
CUY13599	Mat alpha *leu2-3,112 ura3-52 his3-Δ200 trp1-Δ901 lys2-801 suc2-Δ9 VPS11-1::HIS3-HA VPS8::ALFA-HphNT1*	This study
CUY13558	Mat alpha *leu2-3,112 ura3-52 his3-Δ200 trp1-Δ901 lys2-801 suc2-Δ9 VPS8::ALFA-HphNT1*	This study
CUY13560	Mat alpha *leu2-3,112 his3-Δ200 trp1-Δ901 lys2-801 suc2-Δ9 VPS8::ALFA-HphNT1*	This study

Supplementary Table 2. Oligonucleotides used in the study.

Primer	Sequence	used for
S1 Vps3	cagtcaaggagactacctttttttggttgcaaccataatattatagaaccgaattcgagctcgtttaaac	N-terminal tagging with Gal1 promotor
S4 Vps3	ccttcattttcctttacttcttttcctttgtcattattcgttttcttttttaccattttgagatccgggtttt	N-terminal tagging with Gal1 promotor
S1 Vps8	ggcttaataagtgtaaaatatatatctgccgagaccattactcattacacctagagaattcgagctcgtttaaac	N-terminal tagging with Gal1 promotor
S4 Vps8	gtcgtatcgatgctagatctgctgtcgtggtcaaggccattttgctccattttgagatccgggtttt	N-terminal tagging with Gal1 promotor
S1 Vps11	agttaaaccttccaaaaatcatagcgtttcatctataggcacagcaaatcagtcgtacgctgcaggtcgag	N-terminal tagging with Gal1 promotor
S4 Vps11	cttatgggaatattctcgaaaagctggaattgcctccaggagctcagggacatcgatgaattctctgtct	N-terminal tagging with Gal1 promotor
S1 Vps16	gaatagtacagacggataggcctcccttttgtttcgcactaataaaaatgcgtacgctgcaggtcgac	N-terminal tagging with Gal1 promotor
S4 Vps16	ctataaaatacgtctttcaatctttcccagtcgaagctagggtttttcatcgatgaattctctgtcg	N-terminal tagging with Gal1 promotor
S1 Vps18	aaaaactataaggtaccaagaagtaaaaagagaaatatagggatataatgcgtacgctgcaggtcgac	N-terminal tagging with Gal1 promotor
S4 Vps18	gtattccctgtgaggaattgtaactgaacttcctctatacgtgtttttatcatcgatgaattctctgtcg	N-terminal tagging with Gal1 promotor
S1 Vps33	gaaaaagctgatattgcccatctcaaactttatcaaatcatttcacgatgcgtacgctgcaggtcgac	N-terminal tagging with Gal1 promotor
S4 Vps33	gtccatcggcatttgttaatgaaaatttcttagtattccaaaatctattcatcgatgaattctctgtcg	N-terminal tagging with Gal1 promotor
S2 Vps8	tataaattttacttttatgtaaccaaagttgtattaaatatttagaaatgatcgatgaattcgagctcg	C-terminal tagging with FLAG or ALFA tag
S3 Vps8	atgaatattcttgtttaatttgccagacggaatctaacccaaaaatagtacgtacgctgcaggtcgac	C-terminal tagging with FLAG or ALFA tag
S2 Vps18	ccctcttttaatttcagtgtttcagcctgactaaaaaagaatactaatcgatgaattcgagctcg	C-terminal tagging with FLAG tag
S3 Vps18	cagccaatatctattgatgaaacagaattagccaaatggaatgaacgtacgctgcaggtcgac	C-terminal tagging with FLAG tag
S4 Vps18 aa336-end	aatttgttatcccgaactaataaattccataccgaattaggctcattttccattttgagatccgggtttt	Truncation of Vps18 N-Terminus
S4 Vps11 aa349-end	tccttttggatgatgatgttaatttgattttctaaagatttcatatcatcgatgaattctctgtcg	Truncation of Vps11 N-Terminus
S2 Mup1	gttcatacgtgattataagaatcgagatgagatggtaagtacctttttggttaatcgatgaattcgagctcg	C-terminal tagging of Mup1
S3 Mup1	cgttattgaaacgaatataatcgaacattacaaaagtgaacaagaaaaatcgctgcgtacgctgcaggtcgac	C-terminal tagging of Mup1

Supplementary Table 3. Cryo-EM data collection, refinement and validation statistics.

	CORVET composite map (EMD-18701) (PDB 8QX8)	CORVET consensus map (EMD-18707)	CORVET Vps8-Vps11 local (EMD-18702)	CORVET SNARE binding module local (EMD-18704)	CORVET Vps8 β-propeller local (EMD-18703)	CORVET core local (EMD-18705)	CORVET Vps18 β-propeller local (EMD-18706)	CORVET Vps11ΔN mutant (EMD-18708)
**Data collection and processing**								
Magnification	130,000							
Voltage (kV)	200							
Electron exposure (e–/Å^2)	50							
Defocus range (μm)	-0.8 to -2.8							
Pixel size (Å)	0.924							
Symmetry imposed	C1							
Initial particle images (no.; after duplicates removal)	1,445,679							245,790
Final particle images (no.)	219,391	219,391	218,807	211,131	219,391	218,807	219,391	75,213
Map resolution (Å)	3.8-4.6	4.5	4.2	3.8	4.4	4.3	4.6	8.5
FSC threshold	0.143	0.143	0.143	0.143	0.143	0.143	0.143	0.143
**Refinement**								
Initial model used	AlphaFold/ PDB:7ZU0	-	-	-	-	-	-	-
Model resolution range (Å)	3.8-4.6	-	-	-	-	-	-	-
FSC threshold	0.143							
Map sharpening *B* factor (Å^2)	-	133.6	95.9	95.6	89.2	118.1	138.1	754.5
Model composition								
Non-hydrogen atoms	30231	-	-	-	-	-	-	-
Protein residues	4468							
R.m.s. deviations								
Bond lengths (Å)	0.002	-	-	-	-	-	-	-
Bond angles (°)	0.554							
Validation								
MolProbity score	2.03	-	-	-	-	-	-	-
Clashscore	8.74							
Poor rotamers (%)	0							
Ramachandran plot								
Favored (%)	89.68	-	-	-	-	-	-	-
Outliers (%)	0.07							

Supplementary References

1. Ostrowicz, C. W. *et al*. Defined Subunit Arrangement and Rab Interactions Are Required for Functionality of the HOPS Tethering Complex. *Traffic* 11, 1334–1346 (2010).

2. Robinson, J.S. *et al*. A putative zinc finger protein, Saccharomyces cerevisiae Vps18p, affects late Golgi functions required for vacuolar protein sorting and efficient alpha-factor prohormone maturation. *Mol Cell Biol* 11(12), 5813-5824 (1991).

3. Shvarev, D. *et al*. Structure of the HOPS tethering complex, a lysosomal membrane fusion machinery. *eLife* vol. 11 e80901. 13 Sep. 2022, doi:10.7554/eLife.8090
