# Supplementary material for: Structure of the endosomal CORVET tethering complex
Source: Nat Commun. 2024 Jun 19;15:5227. doi: 10.1038/s41467-024-49137-9 (PMC11187117; doi:10.1038/s41467-024-49137-9)
Supplement: Supplementary file 3 — Description of Additional Supplementary Files [file 41467_2024_49137_MOESM3_ESM.pdf]

Description of Additional Supplementary Files

File Name: Supplementary Movie 1

Description: Overall architecture of CORVET tethering complex. Movie representing transition between molecular surface and ribbon representation of the structure. Coloring as in Figure 1.

File Name: Supplementary Movie 2

Description: 3D variability of CORVET. Movie shows eigenvectors of variability in the dataset (variability component 000, also see Supplementary Fig. 5).

File Name: Supplementary Movie 3

Description: 3D variability of CORVET. Movie shows eigenvectors of variability in the dataset (variability component 001, also see Supplementary Fig. 5).

File Name: Supplementary Movie 4

Description: 3D variability of CORVET. Movie shows eigenvectors of variability in the dataset (variability component 002, also see Supplementary Fig. 5).
