# Supplementary material for: Structure of the endosomal CORVET tethering complex
Source: Nat Commun. 2024 Jun 19;15:5227. doi: 10.1038/s41467-024-49137-9 (PMC11187117; doi:10.1038/s41467-024-49137-9)
Supplement: Supplementary file 8 — Reporting Summary [file 41467_2024_49137_MOESM8_ESM.pdf]

nature portfolio

Corresponding author(s): Christian Ungermann, Arne Moeller

Last updated by author(s): Apr 29, 2024

Reporting Summary

Nature Portfolio wishes to improve the reproducibility of the work that we publish. This form provides structure for consistency and transparency in reporting. For further information on Nature Portfolio policies, see our Editorial Policies and the Editorial Policy Checklist.

Statistics

For all statistical analyses, confirm that the following items are present in the figure legend, table legend, main text, or Methods section.

n/a	Confirmed	
☐	☒	The exact sample size (*n*) for each experimental group/condition, given as a discrete number and unit of measurement
☒	☐	A statement on whether measurements were taken from distinct samples or whether the same sample was measured repeatedly
☐	☒	The statistical test(s) used AND whether they are one- or two-sided<br>*Only common tests should be described solely by name; describe more complex techniques in the Methods section.*
☒	☐	A description of all covariates tested
☒	☐	A description of any assumptions or corrections, such as tests of normality and adjustment for multiple comparisons
☐	☒	A full description of the statistical parameters including central tendency (e.g. means) or other basic estimates (e.g. regression coefficient) AND variation (e.g. standard deviation) or associated estimates of uncertainty (e.g. confidence intervals)
☐	☒	For null hypothesis testing, the test statistic (e.g. *F*, *t*, *r*) with confidence intervals, effect sizes, degrees of freedom and *P* value noted<br>*Give P values as exact values whenever suitable.*
☒	☐	For Bayesian analysis, information on the choice of priors and Markov chain Monte Carlo settings
☒	☐	For hierarchical and complex designs, identification of the appropriate level for tests and full reporting of outcomes
☒	☐	Estimates of effect sizes (e.g. Cohen's *d*, Pearson's *r*), indicating how they were calculated

Our web collection on statistics for biologists contains articles on many of the points above.

Software and code

Policy information about availability of computer code

Data collection	EPU 3.7.1. was used for cryo-EM data collection. SoftMaxPro 7.0.2 was used for the acquisition of tethering data. Unicorn 7.6 was used for size exclusion chromatography. Refeyn AcquireMP v2023 was used for mass photometry data collection. Zen 3.2 blue edition was used for light microscopy data collection. Q ExativePlus mass spectrometer was used for the acquisition of mass spectrometry data.
Data analysis	cryoSPARC v3 and v4 was used for cryo-EM data analysis, UCSF ChimeraX 1.7.1, Coot 0.9.6, Phenix 1.20 were used for cryo-EM data analysis and figure preparation, ImageJ 1.52k was used for analysis of negative stain EM data. Origin 2023 (10.0) was used for functional analysis of CORVET-mediated membrane tethering, Refeyn DiscoverMP v2023 was used for mass photometry data analysis. Fiji 1.54-f was used for light microscopy data analysis. MaxQuant v2.0.3.0 was used to analyse mass spectrometry data obtained from pulldown experiments.

For manuscripts utilizing custom algorithms or software that are central to the research but not yet described in published literature, software must be made available to editors and reviewers. We strongly encourage code deposition in a community repository (e.g. GitHub). See the Nature Portfolio guidelines for submitting code & software for further information.

Data

Policy information about availability of data

All manuscripts must include a data availability statement. This statement should provide the following information, where applicable:

- Accession codes, unique identifiers, or web links for publicly available datasets
- A description of any restrictions on data availability
- For clinical datasets or third party data, please ensure that the statement adheres to our policy

The cryo-EM density maps and corresponding atomic models reported in this study have been deposited in the Electron Microscopy Data Bank and Protein Data Bank with the accession codes EMD-18701, -18702, -18703, -18704, -18705, -18706, -18707, -18708 and PDB-8QX8, respectively.
The previously published structure of HOPS tethering complex with the accession code PDB-7ZU0 has been used in this study.

Research involving human participants, their data, or biological material

Policy information about studies with human participants or human data. See also policy information about sex, gender (identity/presentation), and sexual orientation and race, ethnicity and racism.

Reporting on sex and gender	n/a
Reporting on race, ethnicity, or other socially relevant groupings	n/a
Population characteristics	n/a
Recruitment	n/a
Ethics oversight	n/a

Note that full information on the approval of the study protocol must also be provided in the manuscript.

Field-specific reporting

Please select the one below that is the best fit for your research. If you are not sure, read the appropriate sections before making your selection.

☒ Life sciences ☐ Behavioural & social sciences ☐ Ecological, evolutionary & environmental sciences

For a reference copy of the document with all sections, see nature.com/documents/nr-reporting-summary-flat.pdf

Life sciences study design

All studies must disclose on these points even when the disclosure is negative.

Sample size	For tethering assays, tree independent measurements were conducted for every condition tested.<br>In Mup1 uptake assays, 100 cells were quantified for every experimental condition.<br>Sample sizes for biochemical assays were based on accepted standards in the field and deemed sufficient as all attempts at reproduction were successful with the same trend.<br>In cryo-EM experiments, 8.7 millions particles from 33882 micrographs were picked initially for the wt CORVET sample and 2.7 millions particles from 5125 micrographs for the Vps11deltaN CORVET mutant sample. Calculation of micrograph number was not carried out and was defined by the number of collected particles images resulted in the final reconstructions.<br>Growth tests of yeast strains were performed in triplicates using three independend grown cultures which were used for dilutions and spottings (wt, Vps11deltaN, Vps18deltaN).<br>Mass spectrometry experiments were performed in triplicates, starting from three independend yeast cultures, which were analyzed in pulldown experiments (wt, vps11-1, vps11.3 and vps18.1).
Data exclusions	No data were excluded
Replication	All attempts at replication were successful. All experiments were performed three times, independently.
Randomization	For tethering experiments, measurements of different samples were performed in the same imaging conditions. Other randomization was not incorporated into the design of the experiments. All experiments were performed under controlled conditions e.g. temperature, growth phase, incubation times.
Blinding	Experimental blinding was deemed unnecessary since distinct experimental groups were not utilized

Reporting for specific materials, systems and methods

We require information from authors about some types of materials, experimental systems and methods used in many studies. Here, indicate whether each material, system or method listed is relevant to your study. If you are not sure if a list item applies to your research, read the appropriate section before selecting a response.

Materials & experimental systems

n/a	Involved in the study
☒	☐ Antibodies
☐	☒ Eukaryotic cell lines
☒	☐ Palaeontology and archaeology
☒	☐ Animals and other organisms
☒	☐ Clinical data
☒	☐ Dual use research of concern
☒	☐ Plants

Methods

n/a	Involved in the study
☒	☐ ChIP-seq
☒	☐ Flow cytometry
☒	☐ MRI-based neuroimaging

Eukaryotic cell lines

Policy information about cell lines and Sex and Gender in Research

Cell line source(s)	Saccharomyces cerevisiae
Authentication	PCR and sequencing analyses were used for cell line authentication.
Mycoplasma contamination	Cell lines were not tested for mycoplasma contamination.
Commonly misidentified lines (See ICLAC register)	n/a

Plants

Seed stocks	n/a
Novel plant genotypes	n/a
Authentication	n/a
